# PPAR-γ Regulates Carnitine Homeostasis and Mitochondrial Function in a Lamb Model of Increased Pulmonary Blood Flow

**DOI:** 10.1371/journal.pone.0041555

**Published:** 2012-09-04

**Authors:** Shruti Sharma, Xutong Sun, Ruslan Rafikov, Sanjiv Kumar, Yali Hou, Peter E. Oishi, Sanjeev A. Datar, Gary Raff, Jeffrey R. Fineman, Stephen M. Black

**Affiliations:** 1 Vascular Biology Center, Georgia Health Sciences University, Augusta, Georgia, United States of America; 2 Department of Pediatrics, University of California San Francisco, San Francisco, California, United States of America; 3 Cardiovascular Research Institute, University of California San Francisco, San Francisco, California, United States of America; 4 Department of Surgery, University of California Davis, Davis, California, United States of America; Wayne State University, United States of America

## Abstract

**Objective:**

Carnitine homeostasis is disrupted in lambs with endothelial dysfunction secondary to increased pulmonary blood flow (Shunt). Our recent studies have also indicated that the disruption in carnitine homeostasis correlates with a decrease in PPAR-γ expression in Shunt lambs. Thus, this study was carried out to determine if there is a causal link between loss of PPAR-γ signaling and carnitine dysfunction, and whether the PPAR-γ agonist, rosiglitazone preserves carnitine homeostasis in Shunt lambs.

**Methods and Results:**

siRNA-mediated PPAR-γ knockdown significantly reduced carnitine palmitoyltransferases 1 and 2 (CPT1 and 2) and carnitine acetyltransferase (CrAT) protein levels. This decrease in carnitine regulatory proteins resulted in a disruption in carnitine homeostasis and induced mitochondrial dysfunction, as determined by a reduction in cellular ATP levels. In turn, the decrease in cellular ATP attenuated NO signaling through a reduction in eNOS/Hsp90 interactions and enhanced eNOS uncoupling. *In vivo*, rosiglitazone treatment preserved carnitine homeostasis and attenuated the development of mitochondrial dysfunction in Shunt lambs maintaining ATP levels. This in turn preserved eNOS/Hsp90 interactions and NO signaling.

**Conclusion:**

Our study indicates that PPAR-γ signaling plays an important role in maintaining mitochondrial function through the regulation of carnitine homeostasis both *in vitro* and *in vivo*. Further, it identifies a new mechanism by which PPAR-γ regulates NO signaling through Hsp90. Thus, PPAR-γ agonists may have therapeutic potential in preventing the endothelial dysfunction in children with increased pulmonary blood flow.

## Introduction

Carnitine is present in the form of either free carnitine (non-esterified molecule; FC), or acyl carnitines (esterified form; AC). A low AC/FC ratio is indicative of healthy mitochondria whereas a high AC/FC ratio suggests a decreased mitochondrial capacity for energy production. Carnitine and its derivatives are involved in the mitochondrial transport of fatty acids and are critical for the cell to maintain normal mitochondrial function. It is well established that the disruption of carnitine metabolism leads to mitochondrial dysfunction in cells. However, the mechanisms regulating carnitine metabolism are still unresolved. Our previous studies have identified a progressive endothelial dysfunction in a lamb model (Shunt) that mimics congenital heart disease with increased pulmonary blood flow [1]. This correlated with decreased expression and activity of mitochondrial enzymes involved in carnitine metabolism, disrupted carnitine homeostasis, and mitochondrial dysfunction [2].

Peroxisome proliferator-activated receptors (PPARs) are ligand-activated transcription factors that belong to the nuclear hormone receptor family. Recent evidence has established a role for altered PPAR signaling in the development of both systemic and pulmonary vascular disease [3]. PPAR-γ in particular has been shown to play an important role in a number of pathological conditions including diabetes, inflammation, cancer, and atherosclerosis [4], [5]. Recent experimental studies also suggest that loss of PPAR-γ signaling may be involved in the progression of pulmonary hypertension in models of adult [6], [7] and childhood [8] disease. PPAR-γ signaling has been shown to be involved in regulating NO bioavailability [9], [10], [11] although the mechanisms have not been fully elucidated. Prior studies have also indicated a role for PPAR-γ signaling in the regulation of fatty acid metabolism through its ability to modulate the expression of genes involved in the carnitine pathway [12], [13], [14], [15]. As our recent studies have shown that disruptions in carnitine homeostasis [2] correlate with a decrease in PPAR-γ expression [8] and increased NOS-uncoupling [2], this study was designed to determine if the attenuated PPAR-γ signaling in Shunt lambs is directly linked to the disruption of carnitine homeostasis, mitochondrial dysfunction and reduced NO signaling and to determine if PPAR-γ stimulation can preserve endothelial function in Shunt lambs.

## Methods

### Pulmonary arterial endothelial cell (PAEC) culture

Primary cultures of ovine fetal pulmonary arterial endothelial cells (PAEC) were isolated as described previously [16]. All cultures were maintained in Dulbecco's modified eagle medium (DMEM) supplemented with 10% fetal calf serum (Hyclone, Logan, UT), antibiotics/antimycotic (500 IU Penicillin, 500 µg/ml Streptomycin, 1.25 µg/ml Amphotericin B; MediaTech, Herndon, VA) at 37°C in a humidified atmosphere with 5% CO_2_ and 95% air. Cells were used for experiments between passages 3 and 10, seeded at ∼50% confluence, and utilized when fully confluent. Cells were serum-starved overnight prior to treatment.

### Shear stress

Laminar shear stress was applied using a cone-plate viscometer as described previously [17], [18], [19]. PAEC were exposed to a laminar flow rate of 20 dyn/cm^2^ to represent physiological levels of laminar shear stress in the major human arteries.

### Targeting silencing of PPAR-γ by small-interfering RNA

To silence PPAR-γ gene expression, PAEC were grown in 6-well plates to ∼60% confluence and transfected with optimized concentrations of sheep PPAR-γ small interfering RNA (siRNA) (Santa Cruz Biotechnology, sc-156097) or as a control, a scrambled siRNA (Santa Cruz Biotechnology, sc-37007) with no known homology to any sequences from mouse, rat, or human RNA. Transfections were performed using the HiPerfect transfection reagent (Qiagen, cat # 301705) and 25 nM of the appropriate siRNA. After 48 h, whole cell lysates were prepared and the level of PPAR-γ knockdown confirmed using Western blot analysis. PPAR-γ transfected cells were also treated with Rosiglitazone (10 µM) for 24 h and PPAR-γ binding activity, MitoSOX and mitochondrial membrane potential (MMP) studies were conducted as described below.

### PPAR-γ transcriptional activity assay

The PPAR-γ Transcription Factor Assay kit (Cayman Chemical Company, Ann Arbor, MI) was used according to the manufacturer's protocol.

### Determination of mitochondrial ROS levels

MitoSOX™ Red mitochondrial superoxide indicator (Molecular Probes), a fluorogenic dye for selective detection of superoxide in the mitochondria of live cells was used. The MitoSOX Red reagent is live-cell permeant and is rapidly and selectively targeted to the mitochondria. Once in the mitochondria, MitoSOX Red reagent is oxidized by superoxide and exhibits bright red fluorescence upon binding to nucleic acids. After siRNA mediated PPAR-γ silencing, cells were washed with fresh media, and then incubated in media containing MitoSOX Red (2 µM), for ∼30 min at 37°C in dark conditions. Cells were washed with fresh serum-free media and imaged using fluorescence microscopy at an excitation of 510 nm and an emission at 580 nm. A PC-based imaging system consisting of the following components was used for the fluorescent analyses: an Olympus IX51 microscope equipped with a CCD camera (Hamamatsu Photonics) was used for acquisition of fluorescent images. The average fluorescent intensities (to correct for differences in cell number) were quantified using ImagePro Plus version 5.0 imaging software (Media Cybernetics) as previously published [20].

### Analysis of mitochondrial membrane potential

Mitochondrial membrane potential was analyzed using the DePsipher mitochondrial potential assay kit (Trevigen, Gaithersburg, MD) as previously described [21], [22]. Briefly, cells were washed with fresh media, and then the lipophilic cation 5,5′6,6′-tetrachloro-1,1′,3,3′-tetraethyl benzimidazolyl carbocyanine iodide (10 µg/ml) was added. The samples were then incubated for a further 20 min. After an additional wash with Dulbecco's PBS, the red multimeric form within healthy mitochondria was quantified by fluorescence microscopy at 530 nm emission.

### Determination of mitochondrial numbers using Mitotracker green

The total mitochondrial numbers in scrambled and PPAR-γ siRNA transfected ovine PAEC were evaluated by fluorescent microscopy and Fluorescence-Activated Cell Sorting (FACS) using Mitotracker green (Invitrogen, cat # M7514). Cells were stained with Mitotracker dye (100 nM) for 30 min at 37°C. Immunofluorescent images were acquired on an Applied Precision, Delta Vision microscope and the data presented as mean fluorescent intensity. For FACS analysis, after Mitotracker dye exposure, the cells were collected by trypsinization, washed twice with cold PBS, and analyzed by flow cytometry. Fluorescence was quantified using a FACS Calibur Flow Cytometer (BD Biosciences). The data was analyzed using the Cell Quest software and presented as total fluorescent intensity (mean fluorescent intensity x total number of cells).

### Determination of cellular ATP levels

ATP levels were estimated using the firefly luciferin-luciferase method (Invitrogen) as previously published [2].

### Real time quantitative (q) RT-PCR

Quantitative RT–PCR using SYBR green I dye for specific detection of double-stranded DNA was employed to determine CPT1, CPT2 and CrAT mRNA levels in scrambled siRNA and PPAR γ siRNA transfected (48 h) PAEC. Total RNA was extracted using the RNeasy kit (Qiagen), and 1 µg total RNA was reverse-transcribed using QuantiTect Reverse Transcription Kit (Qiagen) in a total volume of 20 µl. Primers for CPT1, CPT2, CrAT and β-actin were designed by Primer 3. The sequences were CPT1 Forward, 5′- CGA CTG GTG GGA GGA ATA CA -3′, Reverse, 5′-TGC GTC TGT AAA GCA GGA TG -3′; CPT2 Forward, 5′-TTG TGC CTT CCT TCC TGT CT-3′, Reverse, 5′-GAG GTG TCT GGC CTT GTC AT-3′; CrAT Forward, 5′-GTT CAG CAG GAC CAA GAA GC-3′, Reverse, 5′-TGC AGT GAC GAG TTC CAG AC-3′; β-actin Forward, 5′-GGG AAA TCG TGC GTG ACA TTA AG -3′, Reverse, 5′-TGT GTT GGC GTA AGG TCT TTG -3′. Real-time Quantitative PCR was conducted using a Mx4000 Multiplex Quantitative PCR System (Stratagene), using 2 µl of RT product, 12.5 µl of QuantiTect SYBR Green PCR Master Mix (Qiagen) and primers (400 nM) in a total volume of 25 µl. The following thermocycling conditions were employed: 95°C for 10 min, followed by 95°C for 30 s, 55°C for 60 s and 72°C 30 s for 40 cycles. 2^−ΔΔ*t*^ values were chosen to reflect the number of mRNA molecules using β-actin (housekeeping gene) as an internal control.

### Surgical Preparation

Ten mixed-breed Western pregnant ewes (137–141 days gestation, term = 145 days) were anesthetized with the use of local anesthesia (2% lidocaine hydrochloride), and inhaled anesthesia (1–3% isoflorane). The pregnant horn of the uterus was exposed, followed by the left fetal chest. With the use of side biting vascular clamps, an 8.0 mm Gore-tex® vascular graft (∼2 mm length) (W.L. Gore and Assos., Milpitas, CA) was anastomosed between the ascending aorta and main pulmonary artery with 7.0 proline (Ethicon Inc., Somerville, NJ), using a continuous suture technique. This procedure was previously described in detail [23]. Beginning immediately after birth, lambs were treated with either Rosiglitazone (n = 5, 3 mg/kg/day) or empty gelatin capsules (n = 5, vehicle control). The drug or vehicle capsules were administered once daily for a 4-week period. The number of vehicle capsules administered was increased to match the upwardly adjusted weight-based dosing of Rosiglitazone. Age matched control lambs served as controls. At the end of the protocol, all lambs were killed with a lethal injection of sodium pentobarbital followed by bilateral thoracotomy as described in the NIH Guidelines for the Care and Use of Laboratory Animals. All protocols and procedures were approved by the Committee on Animal Research of the University of California, San Francisco and the Georgia Health Sciences University.

### Western blot analysis

Protein extracts from peripheral lung tissue or PPAR-γ transfected cells were prepared using lysis buffer (50 mM Tris-HCl, pH 7.6, 0.5%Triton X-100, 20% glycerol) containing Halt™ protease inhibitor cocktail (Pierce Laboratories, Rockford, IL). The extracts were then subjected to centrifugation (15,000 g×15 min at 4°C). Supernatant fractions were assayed for protein concentration using the Bradford reagent (Bio-Rad, Richmond, CA) and used for Western blot analyses. Protein extracts (25–50 µg) were separated on Long-Life 4–20% Tris-SDS-Hepes gels (Frenchs Forest, Australia) and electrophoretically transferred to Immuno-Blot™ PVDF membrane (Bio-Rad Laboratories, Hercules, CA). The membranes were blocked with 5% nonfat dry milk in Tris-buffered saline containing 0.1% Tween 20 (TBST). After blocking, the membranes were probed with antibodies to CPT1 (Affinity Bioreagents), CPT2 (Affinity Bioreagents), CrAT (Santa Cruz Biotechnology, Inc.), eNOS (BD Transduction Laboratories), Hsp90 (Santa Cruz Biotechnology, Inc.), and PPAR-γ (Santa Cruz Biotechnology, Inc.). Reactive bands were visualized using chemiluminescence (SuperSignal® West Femto Substrate Kit, Pierce Laboratories, Rockford, IL) on a Kodak 440CF image station (Kodak, Rochester, NY). Band intensity was quantified using Kodak 1D image processing software. Expression of each protein was normalized by reprobing with ß-actin used as an equal loading control (Sigma, St.Louis, MO).

### Measurement of CrAT activity

Peripheral lung tissue or PPAR-γ transfected cells were lysed in 50 mM Tris-HCl (pH 7.5), 2 mM EDTA, 5 mM MgCl_2_, 0.8 mM DTT, and 0.25 mM PMSF with protease inhibitor cocktail. Samples were briefly sonicated and centrifuged at 3000× *g* for 5 min. CrAT activity was then determined using a modification of the method described by Liu et al [24]. The assay mixture consisted of 50 mM Tris-HCl (pH 7.5), 2 mM EDTA, 25 mM malate, 0.25 mM NAD, 12.5 µg/ml rotenone, 0.04% Triton X-100, 12.5 µg/ml malic dehydrogenase, 17.5 µg/ml citrate synthase, 400 µM CoA, and 2 mM ALCAR. Each sample with a protein concentration between 0.5 mg/ml and 1.0 mg/ml was added to the assay mixture and reactions were monitored at room temperature for 3 minutes at 340 nm absorbance using a Shimadzu Pharmaspec UV-1700 spectrophotometer. The nmoles of product formed was calculated using the extinction coefficient of NADH.

### Measurement of Carnitine homeostasis

For free carnitine (L-carnitine and acetyl L-carnitine) determination, 100 µl homogenates, 300 µl water and 100 µl of internal standard (Sigma ST1093) were mixed. For total carnitine determination 100 µl homogenates were hydrolyzed with 0.3 M KOH, heated at 45°C, pH neutralized using perchloric acid, the volume was made to 400 µl and 100 µl internal standard was added. All samples were purified using solid phase extraction columns, SAX 100 mg/ml (Varian, Harbor City, CA) and derivatized using aminoanthracene in presence of EDCI (catalyst) and kept at 30°C for 1 hour to complete reaction of carnitines. Separation was carried out with an isocratic elution in 0.1 M Tris-acetate buffer (pH 3.5): acetonitrile (68∶32, v/v) at a flow rate of 0.9 ml/min. Detection of carnitines was performed using a Shimadzu UFLC system with a 5 µm Omnispher C18 column (250×4.6 mm OD) and equipped with an RF-10AXL fluorescence detector (Shimadzu USA Manufacturing Corporation). Total and free carnitine levels were quantified by fluorescence detection at 248 nm (excitation) and 418 nm (emission). Acyl carnitines were calculated as total carnitine minus free carnitine as previously published [2].

### Immunoprecipitation analyses

PAEC were lysed in ice cold lysis buffer containing 1% Triton X-100, 20 mM Tris, pH 7.4, 100 mM NaCl, 1 mM EDTA, 1% sodium deoxycholate, 0.1% SDS supplemented with protease inhibitor cocktail (Pierce Laboratories, Rockford, IL). Insoluble proteins were removed by centrifugation at 14,000 rpm for 15 min at 4°C and supernatants were incubated with anti-eNOS antibody (BD Transduction Laboratories) overnight at 4°C and then with protein G plus/protein A agarose suspension (Calbiochem, La Jolla, CA) for 2 h at 4°C for eNOS/Hsp90 immunoprecipitation studies. The immune complexes were precipitated by centrifugation, washed three times with the lysis buffer and boiled in SDS sample buffer for 5 min. Agarose beads were pelleted by centrifugation, and protein supernatants were loaded and run on (4–20%) polyacrylamide gels followed by transfer of the proteins to nitrocellulose membranes. Membranes was blocked with 2% BSA in Tris-buffered saline containing 0.05% Tween 20 (TBST) for 2 h at room temperature, incubated with an anti-Hsp90 antibody (BD transduction Laboratories) for 2 h at room temperature, washed three times with TBST (room temperature, 10 min), then incubated with an HRP conjugated secondary antibody (Pierce Laboratories, Rockford, IL). The reactive bands were visualized with the SuperSignal West Femto Maximum Sensitivity Substrate Kit (Pierce Laboratories, Rockford, IL) and Kodak 440CF image station (Kodak, New Haven, CT). The same blot was re-probed with anti-eNOS antibody to normalize for the levels of immunoprecipitated-eNOS in each sample or re-probed with anti-Hsp90 to normalize for total Hsp90 levels.

### Measurement of NOS-derived superoxide levels in PAEC and lung tissue

Superoxide levels in PPAR-γ siRNA transfected endothelial cells subjected to shear and lung tissues from control, shunt and rosiglitazone-treated shunt lambs, were estimated by electron paramagnetic resonance (EPR) assay using the spin-trap compound 1-hydroxy-3-methoxycarbonyl-2,2,5,5-tetramethylpyrrolidine HCl (CMH, Axxora) as described previously [25], [26]. Superoxide in PAEC was trapped by incubating PAEC with 20 µl of CMH stock solution (20 mg/ml) for 1 h, followed by trypsinization and centrifugation at 500 g. The cell pellet was suspended in 35 µl DPBS and loaded into a capillary tube which was then analyzed with a MiniScope MS200 EPR machine (Magnettech, Berlin, Germany). Freshly frozen lung tissues were pulverized (10–15 mg) and then, homogenized in 150 µl of EPR buffer for 30 s for measurement of superoxide. Sample volumes were then adjusted with 20 mg/ml CMH hydrochloride to achieve final CMH concentration of 10 mg/ml. 35 µl of supernatant was loaded onto a capillary tube and analyzed using the EPR machine. NOS-derived superoxide was measured by pre-incubating tissue lysate with 100 µM ethylisothiourea (ETU, Sigma-Aldrich) for 30 min followed by incubation with CMH. EPR spectra were analyzed using ANALYSIS v.2.02 software (Magnettech). Differences between levels of samples incubated in the presence and absence of ETU were used to determine NOS-dependant superoxide generation. eNOS-dependent superoxide levels were reported as nmols/min/mg protein.

### Measurement of NO_x_ levels in PAEC and lung tissue

To measure NO_x_ level in tissue samples we utilized a chemiluminescence method. Samples were deproteinized by adding cold ethanol (1∶4; v/v). 0.05 g of potassium iodide (KI) was dissolved in 7 ml of freshly prepared acetic acid (AA). The KI/AA reagent was added to a septum sealed purge vessel and bubbled with nitrogen gas. The gas stream was connected via a trap containing 1N NaOH to a Sievers 280i Nitric Oxide Analyzer (GE). Deproteinized samples were injected with a syringe through a silicone/Teflon septum. Results were analyzed by measuring the area under curve of the chemiluminescence signal using the Liquid software (GE).

### Determination of lactate and pyruvate levels

Lung tissues were homogenized in ice-cold 0.5 M perchloric acid then centrifuged at 14,000 rpm for 20 min. The supernatants were then neutralized with 3 M KHCO_3_ and used for lactate and pyruvate assays. The relative changes in lactate levels were measured by using lactate assay kit (Biovision). Pyruvate levels were determined using the spectrophotometric enzymatic measurement assay at 340 nm. NADH was used as a cofactor and lactate dehydrogenase (LDH) as the co-enzyme. Experimental conditions were as previously published [27].

### Statistical analyses

This was performed using GraphPad Prism version 4.01 for Windows (GraphPad Software, San Diego, CA). The mean ± SEM were calculated for all samples and significance was determined either by the unpaired t-test (for 2 groups) or by ANOVA (for ≥3 groups). When the data was non-normally distributed, non-parametric testing was used (Wilcoxon signed-rank for 2 groups, and the Kruskal-Wallis test for ≥3 groups). A value of p<0.05 was considered significant.

## Results

### PPAR-γ siRNA significantly decreases PPAR-γ expression and activity in pulmonary arterial endothelial cells

**Figure 1:**
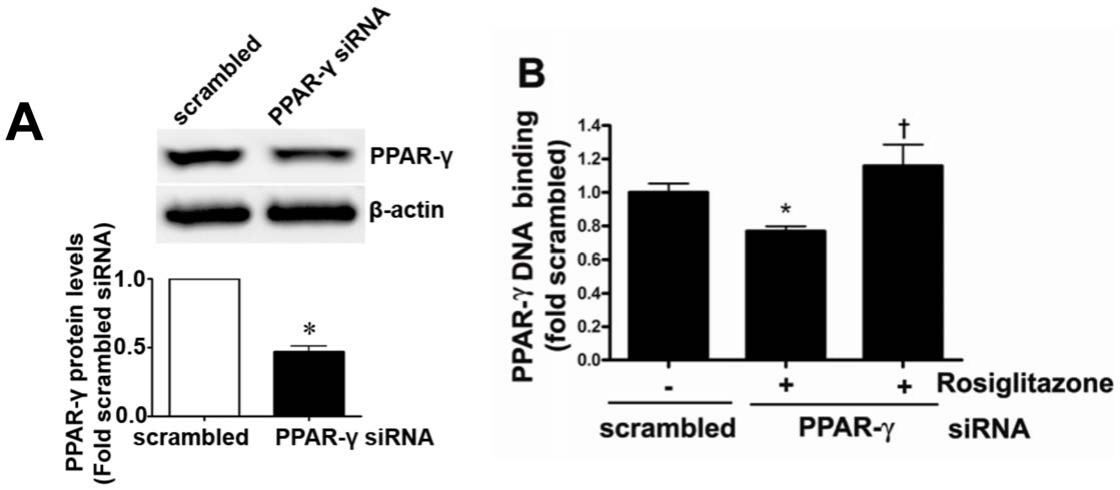
PPAR-γ gene silencing decreases PPAR-γ protein levels and activity in ovine pulmonary arterial endothelial cells. Protein extracts (20 µg) prepared from PAEC transfected with a PPAR-γ siRNA or a scrambled control for 48 h, were analyzed by Western blot analysis and a significant decrease in PPAR-γ protein levels was observed (A). The decrease in PPAR-γ binding activity was reversed after treatment with PPAR-γ agonist, rosiglitazone (10 µM; 24 h) (B). Values are mean ± SE; n = 6–12. *P<0.05 *vs* scrambled siRNA, †P<0.05 *vs* PPAR-γ siRNA.

PAEC were transiently transfected with a PPAR-γ siRNA or a scrambled siRNA and the level of PPAR-γ knockdown was confirmed using Western blot analysis. There was a significant (>50%) reduction in PPAR-γ protein levels in PPAR-γ siRNA transfected cells (Figure 1 A). PPAR-γ binding activity was also significantly decreased in PPAR-γ siRNA transfected cells, and this was reversed after treatment with the PPAR-γ agonist, rosiglitazone (Figure 1 B).

### Attenuating PPAR-γ signaling disrupts carnitine homeostasis in pulmonary arterial endothelial cells

**Figure 2:**
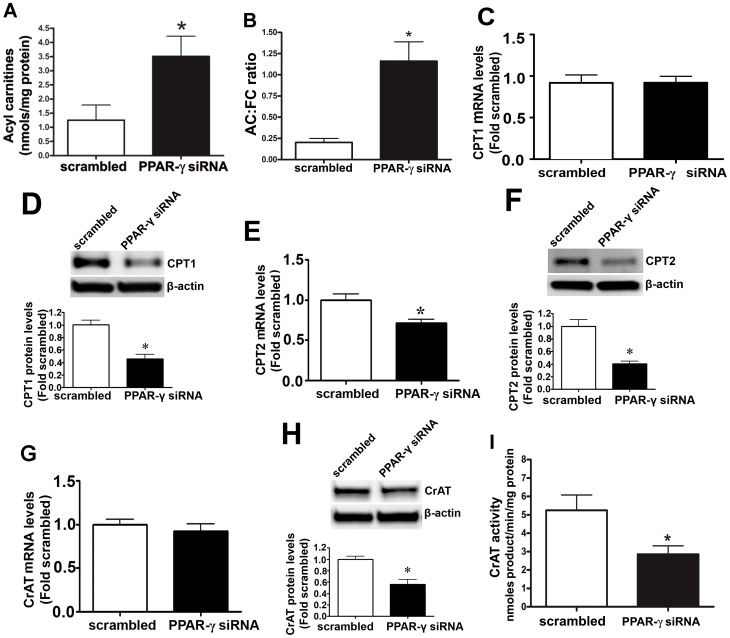
Inhibition of PPAR-γ signaling disrupts carnitine homeostasis in ovine pulmonary arterial endothelial cells. PAEC were transiently transfected with a PPAR-γ siRNA or a scrambled siRNA for 48 h. HPLC analysis was then performed to determine cellular carnitine levels. There was a significant increase in both acyl carnitine levels (A) and the acyl carnitine to free carnitine (AC∶FC) ratio (B) in PPAR-γ siRNA transfected cells, indicating disruption of carnitine homeostasis. Western blot analysis identified a significant decrease in CPT1 (D), CPT2 (F), and CrAT (H) protein levels in the PPAR-γ siRNA transfected cells. The decrease in CrAT protein levels correlated with a significant decrease in CrAT activity (I). The mRNA levels of CPT2 (E) decreased significantly whereas there was no change in CPT1 (C) and CrAT (G) mRNA levels. Values are mean ± SE; n = 5–12. *P<0.05 *vs* scrambled siRNA.

The effect of reducing PPAR-γ signaling on cellular carnitine homeostasis was determined using HPLC analysis. PPAR-γ siRNA transfection led to a significant increase in acyl carnitine levels (Figure 2 A) and an increased AC/FC ratio (Figure 2 B). Further, the disruption in carnitine homeostasis correlated with a significant decrease in CPT1- (Figure 2 D), CPT2- (Figure 2 F), and CrAT (Figure 2 H)-protein levels as well as CrAT activity (Figure 2 I). Further, there was a significant decrease in CPT2 mRNA levels (Figure 2 E) but CPT1 and CrAT mRNA levels did not change (Figure 2 C & G).

### Disruption of carnitine homeostasis disrupts mitochondrial function, decreases eNOS/Hsp90 interactions and causes eNOS uncoupling

**Figure 3:**
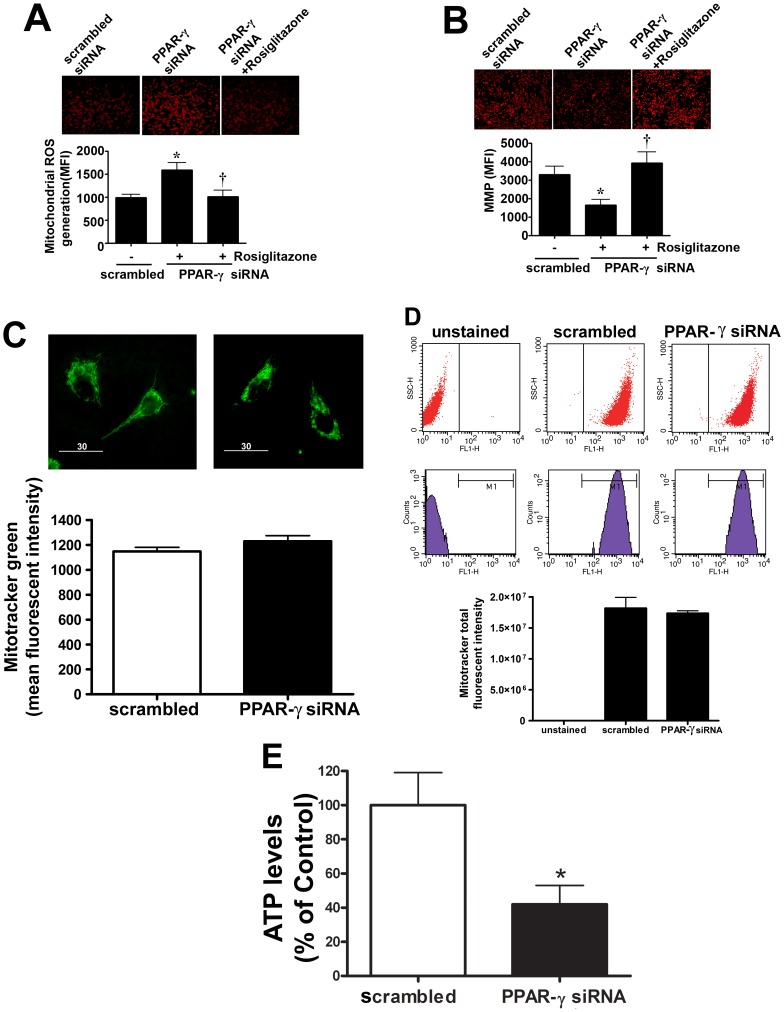
Decreased PPAR-γ signaling induces mitochondrial dysfunction in ovine pulmonary arterial endothelial cells. PAEC were transiently transfected with a PPAR-γ siRNA or a scrambled siRNA for 24 h then exposed or not to the PPAR-γ agonist, rosilglitazone (10 µM) for a further 24 h. The MitoSOX red mitochondrial ROS indicator was then added. Representative images after MitoSOX staining are shown (A, top). Images of 20 random fields were quantified to determine the mean fluorescence intensity of each sample. PPAR-γ inhibition significantly increased mitochondrial ROS levels and this was reversed by rosiglitazone (A). Mitochondrial membrane potential (MMP) was also determined using the DePsipher mitochondrial potential assay kit. Representative images after DePsipher staining are shown (B, top). PPAR-γ inhibition significantly decreased mitochondrial membrane potential and this was reversed by rosiglitazone (B). Total mitochondrial number was evaluated by fluorescent microscopy (C) and flow cytometry (D) in scrambled and PPAR-γ siRNA transfected PAEC stained with Mitotracker green. PPAR-γ gene silencing had no significant affect on mitochondrial number as evaluated by either method. There was also a significant reduction in ATP levels after PPAR-γ siRNA transfection (E). Values are mean ± SE; n = 7–12. *P<0.05 *vs* scrambled siRNA.

**Figure 4:**
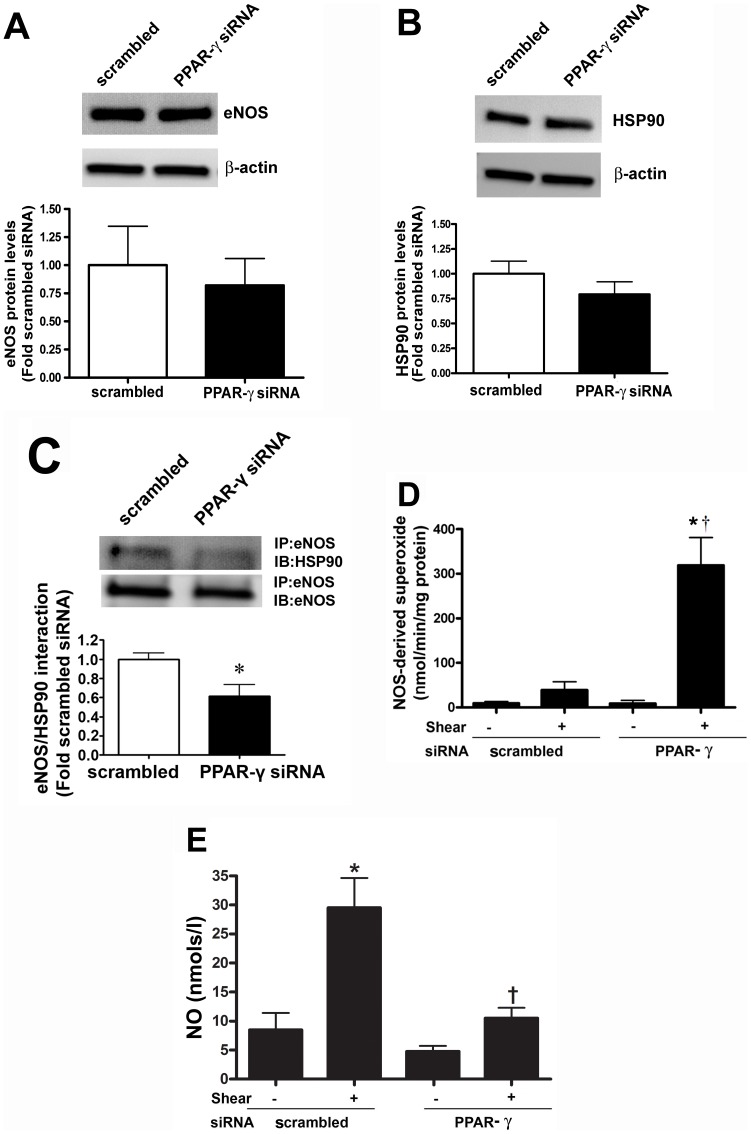
Decreased PPAR-γ signaling increases eNOS uncoupling in ovine pulmonary arterial endothelial cells. The levels of eNOS (A) and Hsp90 (B) protein levels were unchanged between scrambled and PPAR-γ siRNA transfected PAEC. The interaction of eNOS with Hsp90 was determined by immunoprecipitation (IP) using a specific antiserum raised against eNOS followed by Western blot (IB) analysis with an anti-Hsp90 antibody. The membrane was reprobed for eNOS to normalize for immunoprecipitation efficiency. There was a significant decrease in the association of eNOS with Hsp90 in PPAR-γ siRNA transfected cells (C). The effect of PPAR-γ inhibition on eNOS uncoupling was determined. PAEC were treated or not with eNOS inhibitor 2-ethyl-2-thiopseudourea (ETU). Superoxide and NO levels were then determined in the presence or absence of acute laminar shear stress (20dyn/cm^2^, 15 min). PPAR-γ siRNA transfection significantly increased eNOS-derived superoxide levels (D) and decreased NO levels (E) but only under shear-stimulated conditions. Values are mean ± SE; n = 6. *P<0.05 *vs* scrambled siRNA, no shear; †P<0.05 *vs* scrambled siRNA, shear.

As our data indicated that the percentage of carnitine present in its acylated form is increased when PPAR-γ signaling is attenuated, we next examined its effect on mitochondrial function. Suppressing PPAR-γ expression produced a significant increase in mitochondrial ROS levels (Figure 3 A) and mitochondrial membrane potential (Figure 3 B). Treatment with the PPAR-γ agonist, rosiglitazone was able to reverse these changes (Figure 3 A & B). There was no change in the total mitochondrial numbers in the scrambled and PPAR-γ siRNA as determined by Mitotracker staining (Figure 3 C) and FACS analysis (Figure 3 D). However, there was a reduction in cellular ATP levels (Figure 3 E), indicating that the loss of carnitine homeostasis induced by the attenuation of PPAR-γ signaling leads to mitochondrial dysfunction. Hsp90 is an ATP dependent chaperone and the interaction of Hsp90 with eNOS has been shown to increase eNOS coupling and NO production [25]. Thus, we next examined the effect of the decrease in cellular ATP levels on eNOS/Hsp90 interactions. We did not find any change in the eNOS and Hsp90 protein levels (Figure 4 A & B) after PPAR-γ gene silencing. However, utilizing immunoprecipitation analyses we found that decreasing PPAR-γ signaling significantly decreased the binding of Hsp90 to eNOS (Figure 4 C). In addition, we found that in response to acute shear stress, eNOS dependent superoxide generation was enhanced (Figure 4 D) while NO production was reduced (Figure 4 E) in PPAR-γ siRNA transfected cells. Basal superoxide production and NO generation were unaffected by PPAR-γ siRNA transfection. Together these data indicate that the impaired mitochondrial function induced by PPAR-γ inhibition leads to uncoupling of eNOS and reduced NO signaling.

### The PPAR-γ agonist, rosiglitazone preserves carnitine homeostasis in lambs with increased pulmonary blood flow

**Figure 5:**
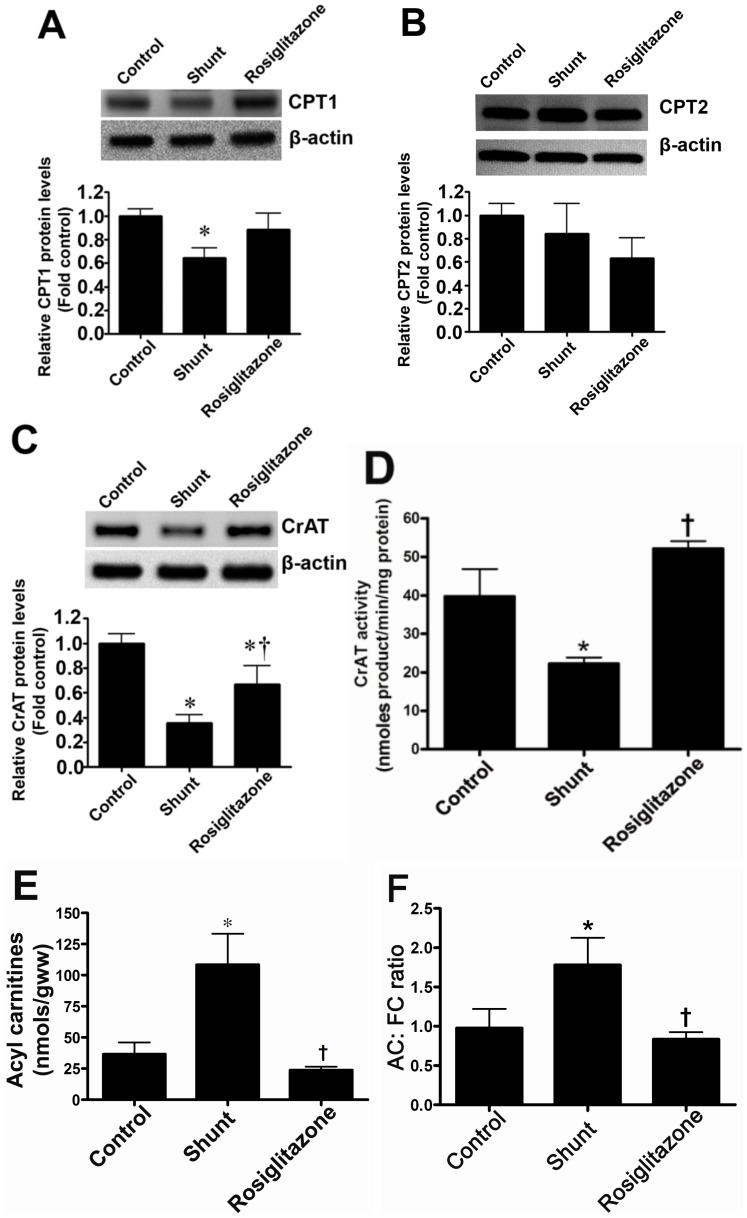
Rosiglitazone treatment preserves carnitine homeostasis in lambs with increased pulmonary blood flow. Protein extracts (50 µg) were prepared from peripheral lung tissues from vehicle- or rosiglitazone-treated Shunt lambs and age-matched controls. CPT1, CPT2 and CrAT protein levels were then determined by Western blot analyses. There was a significant decrease in CPT1 (A) and CrAT (C) protein levels in the vehicle-treated Shunt lambs. Rosiglitazone treatment prevented the decrease in the protein levels of CrAT enzyme (C). There was no significant change in the CPT2 protein levels (B) in any of the 3- groups. There was also a significant decrease in CrAT activity in vehicle-treated Shunt lambs, which was prevented by rosiglitazone (D). Acyl carnitines (E) and the AC∶FC ratio (F) were found to be significantly higher in the vehicle-treated Shunt lambs, indicating disruption of carnitine homeostasis. However, carnitine homeostasis was preserved by rosiglitazone treatment (E & F). Values are mean ± SE; n = 4–6 for each group. *P<0.05 *vs* control; †P<0.05 *vs* shunt.

We have previously shown that both PPAR-γ signaling [8] and carnitine homeostasis are disrupted in Shunt lambs. Thus, we next determined whether the PPAR-γ agonist, rosiglitazone could preserve carnitine homeostasis in these lambs. Compared to age-matched Control lambs, CPT1 (Figure 5 A) and CrAT (Figure 5 C) protein levels are reduced in vehicle-treated Shunt lambs, although CPT2 protein was unchanged (Figure 5 B). Rosiglitazone treatment preserved CPT1 (Figure 5 B) and CrAT protein levels (Figure 5 C) and prevented the decrease in CrAT activity in Shunt lambs (Figure 5 D). Carnitine homeostasis was also preserved (Figure 5 E & F).

### Rosiglitazone preserves mitochondrial function in lambs with increased pulmonary blood flow

**Figure 6:**
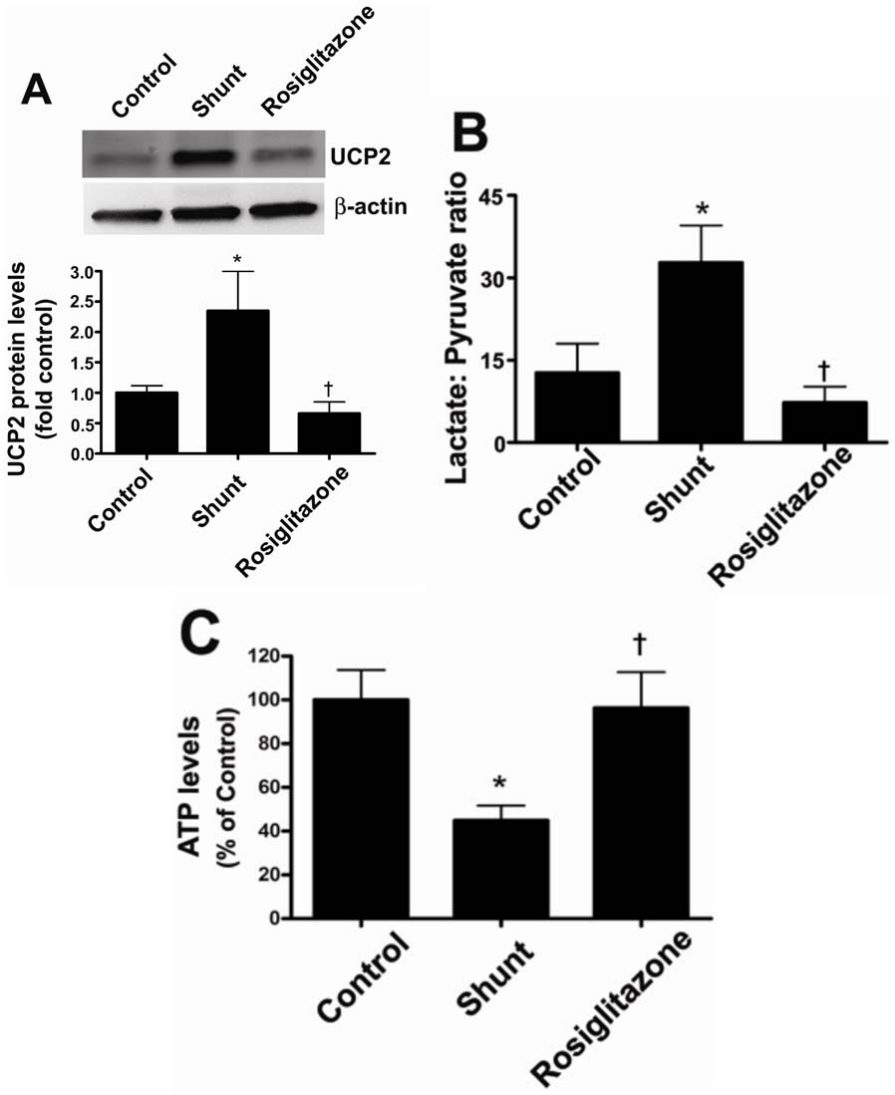
Rosiglitazone treatment preserves mitochondrial function in lambs with increased pulmonary blood flow. Protein extracts (50 µg) were prepared from peripheral lung tissues from vehicle- or rosiglitazone-treated Shunt lambs and age-matched controls. UCP-2 protein levels were then determined using Western blot analysis. There was a significant increase in UCP-2 protein levels in the vehicle-treated Shunt lambs (A). The increase in UCP-2 was not observed in Shunt lambs treated with rosiglitazone (A). There was also a significant reduction in lactate∶pyruvate ratio (B) and ATP levels (C) in vehicle-treated Shunt lambs. However, both the lactate∶pyruvate ratio and ATP levels were preserved by rosiglitazone and were unchanged compared to age-matched control lambs (B & C). Values are mean ± SE; n = 5–7 for each group. *P<0.05 *vs* control; †P<0.05 *vs* shunt.

Shunt lambs have mitochondrial dysfunction, exhibiting significantly higher UCP-2 protein levels (Figure 6 A), a higher lactate/pyruvate ratio (Figure 6 B), and reduced ATP levels (Figure 6 C) compared to age-matched controls. Rosiglitazone treatment prevented the increase in UCP-2 protein levels (Figure 6 A), the disruption of the lactate/pyruvate ratio (Figure 6 B), and the reduction in ATP levels (Figure 6 C) indicative of improved mitochondrial function.

### Rosiglitazone restores eNOS/Hsp90 interactions and NO generation in lambs with increased pulmonary blood flow

**Figure 7:**
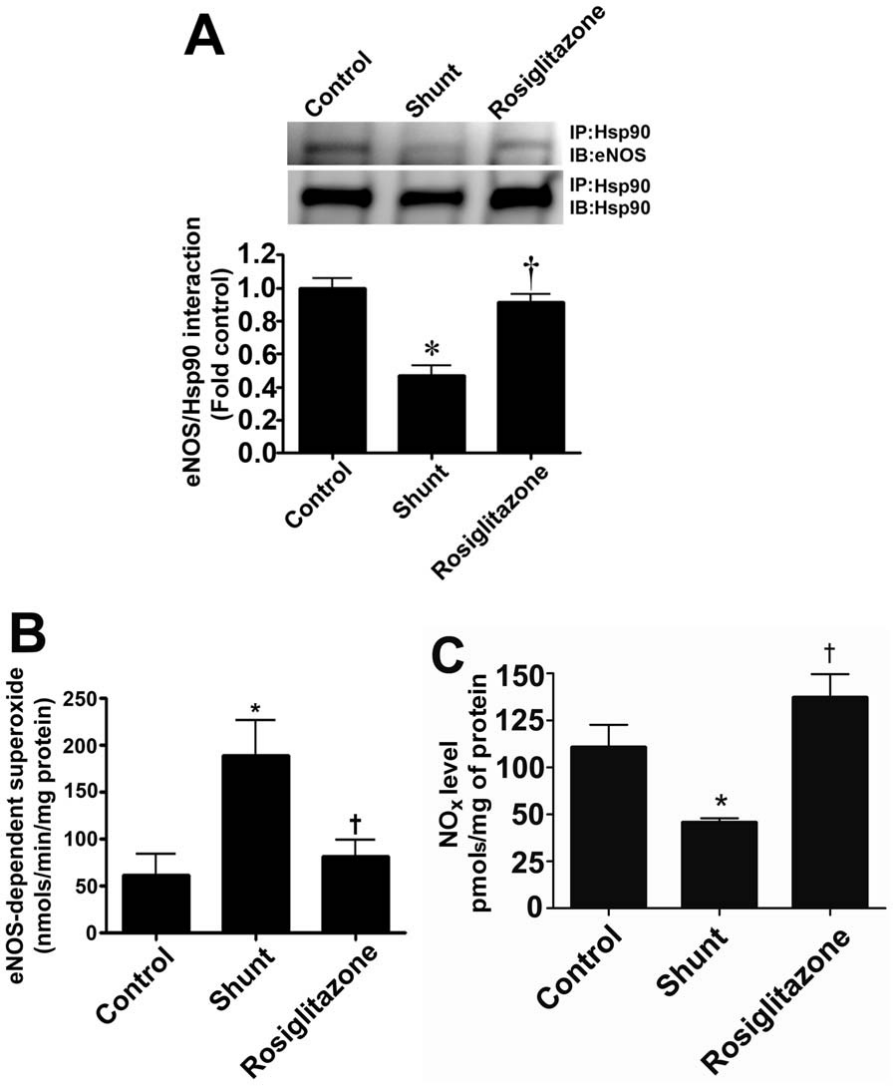
Rosiglitazone treatment preserves NO signaling in lambs with increased pulmonary blood flow. The interaction of eNOS with Hsp90 was determined by immunoprecipitation using specific antiserum raised against eNOS using peripheral lung extracts prepared from vehicle- or rosiglitazone-treated Shunt lambs and age-matched controls. Immunoprecipitated extracts were analyzed using antisera against either eNOS or Hsp90. The levels of eNOS protein associated with Hsp90 relative to total eNOS protein were calculated. There is a significant decrease in the association of eNOS with Hsp90 in vehicle-treated Shunt lambs, which is prevented by rosiglitazone (A). Further, there is an increase in NOS-derived superoxide generation (B) and decreased NO_x_ levels (C) in vehicle-treated Shunt lambs, indicating increased eNOS uncoupling. Rosiglitazone treatment preserved eNOS coupling and both NOS-derived superoxide (B) and NO_x_ (C) were unchanged to observed in age-matched control lambs. Values are mean ± SE; n = 4–8 for each group. *P<0.05 *vs* control; †P<0.05 *vs* shunt.

Our data indicate that the decreased association of eNOS with Hsp90 in Shunt lambs correlating with reduced ATP levels is attenuated by rosiglitazone (Figure 7 A). Further, the increase in eNOS uncoupling (Figure 7 B) and reductions in NO generation (Figure 7 C) in Shunt lambs was prevented by rosiglitazone indicating that endothelial function is preserved.

## Discussion

An expanding body of literature has established the importance of endothelial dysfunction in the pathogenesis of pulmonary vascular disease. However, a consensus on what defines endothelial dysfunction has not emerged. At present a number of factors are used to define this process, including alterations in structure, perturbations in the normal production and/or balance of endothelial-derived factors, and functional abnormalities, including increased constriction and/or impaired relaxation. It is clear that the mechanisms responsible for these signaling and structural changes are multi-factorial and this study provides insight into a novel mechanism by which the attenuation of PPAR-γ signaling reduces NO signaling in PAEC through mitochondrial dysfunction. The important findings of this study are: (i) PPAR-γ inhibition decreases the expression proteins involved in carnitine metabolism resulting in the disruption of carnitine homeostasis; (ii) The disruption in carnitine homeostasis leads to mitochondrial dysfunction and decreased cellular ATP generation; (iii) The decrease in cellular ATP levels disrupts the eNOS-Hsp90 interaction leading to eNOS uncoupling and decreased NO signaling; (iv) The PPAR-γ agonist, rosiglitazone preserves mitochondrial- and endothelial-function in lambs with increased pulmonary blood flow.

PPAR-γ signaling has a major impact on endothelial biology [3], [28], [29] and alterations in PPAR-γ function appear to play a role in the pathogenesis of pulmonary hypertension, diabetes, atherosclerosis, inflammation, and cancer [4], [5], [6]. Prior studies have identified interactions between PPAR-γ and factors believed to be important for the progression of pulmonary vascular disease including NO, endothelin-1, prostacyclin, asymmetric dimethylarginine, insulin, and the bone morphogenetic protein pathway [30], [31], [32], [33], [34], [35], [36], [37]. Earlier studies have identified a decrease in PPAR-γ mRNA [38] and protein expression in lungs from patients with pulmonary hypertension [6] while rosiglitazone, a PPAR-γ agonist, has been shown to attenuate hypoxia-induced pulmonary hypertension, vascular remodeling, and reactive oxygen species generation in the mouse lung [39], [40]. We also recently reported that PPAR-γ protein levels are significantly down-regulated in the lung of our lamb model of pulmonary hypertension secondary to increased pulmonary blood flow (Shunt) [8] and this correlated with the disruption of carnitine homeostasis [2]. As the mitochondrial membrane is impermeable to long-chain fatty acids, carnitine is required for the transportation of fatty acids into the mitochondria so they are available for β-oxidation. Carnitines are also involved in the removal of accumulated toxic fatty acyl-CoA metabolites, and help in buffering the balance between free and acyl-CoA. The carnitine transport system consists of carnitine palmitoyltransferase 1 (CPT1) localized on the outer mitochondrial membrane and carnitine palmitoyltransferase 2 (CPT2) localized on the inner mitochondrial membrane [41]. PPARs have been shown to regulate the entry of fatty acids into the mitochondria by increasing the expression of the CPT1 gene [14]. This study also identified a PPAR response element (PPRE) in the promoter region of the CPT1 gene [14]. Further, the combined activation of PPAR-γ and β-adrenergic receptors in humans results in a beneficial effect on lipid metabolism in subcutaneous fat by increasing the expression of genes required for fatty acid catabolism which include CPT1 [12]. Also a 3-fold increase in PPAR-γ expression in skeletal muscle has been shown to produce a 13-fold change in expression of CPT1 [13]. Organ specific changes in PPAR-γ signaling can also alter systemic carnitine homeostasis as demonstrated in a recent study where PPAR-γ was over-expressed in the colon [42]. Similarly, our results show that PPAR-γ activation via roziglitazone, preserved free carnitine levels and prevented the increase in acylcarnitines, thus improving carnitine homeostasis.

Our data also suggest that the regulation of the carnitine homeostatic enzymes by PPAR-γ is complex. *In vitro*, PPAR-γ gene silencing reduced the levels of CPT1 and CPT2 protein. At the mRNA level only CPT2 was reduced despite a PPAR response element (PPRE) having been identified in the CPT1 gene [14]. However, PPAR's do not always act independently due to their intrinsic ability to form hetero-complexes with other transcription factors that can then modulate their ability to bind to regulatory *cis*-elements. Thus, it is possible that there is a compensatory upregulation of a co-activator, or alternatively, a downregulation of a transcriptional repressor, that preserves CPT1 mRNA levels. We also found that the levels of CrAT protein are decreased both in siRNA expressing PAEC and in Shunt lambs while CrAT levels were preserved by rosiglitazone in Shunt lambs. However, in ovine PAEC, PPAR-γ gene silencing did not reduce CrAT mRNA levels. The gene for CrAT does not appear to have a PPRE sequence [43]. Thus, the down-regulation of CrAT we have observed in response to decreases in PPAR-γ signaling is likely indirect. Taken together we speculate that the reduction in CPT1 and CrAT protein in ovine PAEC, despite no reduction in mRNA levels, may be explained by an increase in their degradation. Indeed, we have recently shown, in both ovine PAEC and Shunt lambs, that mitochondrial dysfunction leads to an increase in the proteasomal degradation of GTP cyclohydrolase I [44], [45]. However, further studies will be required to test this possibility. Further complexity is shown by the fact that although CrAT activity was significantly less in the 4-week Shunt lambs utilized in this investigation, the reduction was only 2-fold compared to the ∼10-fold we observed at 2-weeks of age again suggesting that mechanisms other than PPAR-γ signaling may be involved in regulating CrAT expression and activity. Also, in Shunt lambs CPT1 but not CPT2 protein levels were decreased. Why CPT2 levels are not decreased in Shunt lambs at 4-weeks of age is unclear, as we have previously shown a significant decrease at 2-weeks of age [2]. However, it is possible that factors other than PPAR-γ are also involved in regulating CPT2 expression in the pulmonary vessel. For example there is data suggesting that increases in TGF-ß1 are inversely correlated with CPT expression [46] and our previous studies indicate that TGF-ß1 levels are elevated in Shunt lambs to a greater extent at earlier ages [47].

Increased blood flow exposes the vascular endothelium to increased shear stress that exerts a variety of effects on endothelial structure and function. The application of shear stress to cultured endothelial cells causes the transient activation of many genes including PPAR-γ [48], [49]. In our study we found that PPAR-γ gene silencing significantly attenuated shear-induced NO production. Further, shear-mediated eNOS derived superoxide levels were also increased after PPAR-γ siRNA transfection. Thus, PPAR-γ has an important role in regulating shear-stimulated NO production in EC and the attenuation of PPAR-γ signaling compromises the response of EC to shear conditions producing eNOS uncoupling. This uncoupling appears to be due to the mitochondrial dysfunction associated with the disruption of carnitine homeostasis enzymes induced by the loss of PPAR-γ signaling. Mitochondria regulate the production of ATP which can directly stimulate NO release via the activation of eNOS [50], [51]. However, the levels of ATP can also indirectly stimulate NO signaling through its modulation of the chaperone activity of the 90 kD heat shock protein (Hsp90). Hsp90 binds to eNOS an stimulates its activity following agonist stimulation in endothelial cells [52], [53], [54] by facilitating the calmodulin induced displacement of caveolin-1 from eNOS [55]. Conversely, pharmacological or biochemical disruption of the eNOS/Hsp90 complex adversely affects eNOS activation. The decreased association of eNOS with Hsp90 leads to enhanced eNOS-dependent production of superoxide [56] as we demonstrate here. Indeed our data are in agreement with an earlier study where it was shown that selected PPAR-γ ligands increase NO release from endothelial cells through the stimulation of eNOS-Hsp90 interactions [10]. The ability of each PPAR-γ ligand to stimulate NO was in turn inhibited by the transfection of a PPAR-γ specific siRNA [10]. Further, it has been shown that endothelial PPAR-γ signaling regulates vascular NO production and the disruption of PPAR-γ contributes to oxidative stress and the appearance of endothelial dysfunction *in vivo* [9]. In our study we found that decreases in eNOS-Hsp90 interactions both in EC and in lung tissues, was associated with decreased NO- and increased superoxide- generation. *In vivo*, this decrease in NO signaling and an increase in NOS-derived superoxide will result in endothelial dysfunction. We also found that rosiglitazone supplementation preserved mitochondrial function, eNOS/Hsp90 interactions, and NO signaling. Thus, rosiglitazone decreased the oxidative stress associated with increased pulmonary blood flow, at least in part, by preserving mitochondrial function and eNOS coupling. However, it is worth noting that the lung contains multiple potential sources for superoxide in addition to the mitochondrion and eNOS. These include NADPH oxidase, lipoxygenase, cyclooxygenase, and xanthine oxidase. In addition, superoxide levels relate not only to its generation, but also to its metabolism and studies indicate that PPAR-γ activation can increase superoxide dismutase and catalase expression and activity [57], [58]. However, determining whether rosiglitazone alters the activity of other superoxide generating systems or antioxidant enzymes in the Shunt lambs will require further studies. It is also worth noting that although we used a validated chemiluminescence method of detecting NO, and its metabolic products nitrite and nitrate, which together are termed NO_X_ [59], [60], the *in vivo* measurement of NO is difficult, as NO has a short half-life and reacts rapidly with ROS, oxygen, metals, sulphydryls, disulfides, and hemoglobin [61]. Thus, it is likely that we are underestimating NO generation in the lung.

In conclusion our findings suggest that PPAR-γ can regulate the balance between NO bioavailability and superoxide generation through mechanisms that involve mitochondrial pathways. Inhibition of PPAR-γ in PAEC leads to a disruption in carnitine homeostasis and subsequent mitochondrial dysfunction. This in turn leads to a reduction in eNOS-Hsp90 interactions (secondary to decreased ATP generation) and reduced NO signaling. Rosiglitazone supplementation preserves mitochondrial function, and prevents endothelial dysfunction *in vivo*. Thus, we speculate that stimulating PPAR-γ signaling or enhancing carnitine homeostasis may have therapeutic benefits in conditions of endothelial dysfunction such as pulmonary hypertension, diabetes, and atherosclerosis.
